# The development and validation of a fast and robust dried blood spot based lipid profiling method to study infant metabolism

**DOI:** 10.1007/s11306-014-0628-z

**Published:** 2014-02-11

**Authors:** Albert Koulman, Philippa Prentice, Max C. Y. Wong, Lee Matthews, Nicholas J. Bond, Michael Eiden, Julian L. Griffin, David B. Dunger

**Affiliations:** 1 Medical Research Council Human Nutrition Research, Elsie Widdowson Laboratory, 120 Fulbourn Road, Cambridge, CB1 9NL UK; 2 Department of Paediatrics, Addenbrooke’s Hospital, University of Cambridge, Cambridge, CB2 0QQ UK

**Keywords:** Lipidomics, Dried blood spots, DIMS, FTMS, Infant lipid metabolism

## Abstract

Early life exposures and metabolic programming are associated with later disease risk. In particular lipid metabolism is thought to play a key role in the development of the metabolic syndrome and insulin resistance in later life. Investigative studies of metabolic programming are limited by the ethics and practicalities of sample collection in small infants. Dried blood spots on filter paper, derived from heel pricks are considered as the most suitable option for this age group. We validated a novel lipid profiling method, based on high resolution mass spectrometry to successfully determine the lipid composition of infants using dried blood spots. The spotting and air drying of blood on paper has noticeable effects on many of the lipids, leading to lipid oxidation and hydrolysis, which demand careful interpretation of the obtained data. We compared the lipid profiles from plasma or whole blood samples and the results from dried blood spots to determine if these revealed the same inter-subject differences. The results from dried blood spots were no less reproducible than other lipid profiling methods which required comparatively larger sample volumes. Therefore, lipid profiles obtained from dried blood spots can be successfully used to monitor infancy lipid metabolism and we show significant differences in the lipid metabolism of infants at age 3 versus 12 months.

**Electronic supplementary material:**

The online version of this article (doi:10.1007/s11306-014-0628-z) contains supplementary material, which is available to authorized users.

## Introduction

Lipidomics is commonly applied to study the aetiology of a range of metabolic diseases including the metabolic syndrome and type II diabetes. Multiple studies have shown that specific phosphatidylcholines and sphingolipids levels, as well as the triglyceride profile are associated with relative risk for type II diabetes (Floegel et al. 2012; Rhee et al. 2011). In addition, there is great interest in processes such as metabolic imprinting and programming, and how this influences the predisposition to insulin resistance in later life (Hattersley and Tooke 1999). This is based on associations between early life exposures, including low birth weight and rapid infancy growth, and increased risk for type II diabetes (Harder et al. 2007) and cardiovascular disease in adulthood (Barker et al. 1990) suggesting that modifying the early postnatal environment may have significant impact on later life disease risk.

Most mechanistic studies related to programming have been carried out using rodent models. Thus far it has been difficult to study lipid metabolism in infants and relate this to later health outcomes due to small cohort design and limited blood sampling in this age group. Many current methods used for detailed lipid or fatty acid profiling require considerable volumes of plasma, serum or blood, well surpassing the 30–50 μl per spot that can be collected from a heel prick. Although more blood can be collected from neonates (Escolano-Margarit et al. 2013; Kuipers et al. 2011; Sabel et al. 2009), the heel prick is generally considered as the least invasive, and therefore preferred by both parents and researchers. Dried blood spots have also become a standard biological medium especially in pharmacokinetic studies (Abu-Rabie and Spooner 2009) and in newborn screening, where mass spectrometry MS/MS methods have been developed for detection of inborn errors of metabolism (Chace et al. 1993; Pandor et al. 2004).

We aimed to develop a lipidomics approach that would allow us to rapidly measure the lipid metabolism of infants using dried blood spots. Initially we were skeptical about the application of dried blood spots as a sample format for the study of lipid metabolism. In particular we wanted to determine the stability of blood lipids on paper and to ascertain to what extent lipid oxidation and hydrolysis affect lipid concentrations generally and whether this was specific to particular classes of lipids. The final aim was to use the method on a sub-set of the Cambridge Baby Growth Study (CBGS) to determine the lipid profile of healthy, breast fed infants at 3 and 12 months of age, providing a proof of concept for our approach. This is the first report that uses dried blood spots for lipidomic profiling.

## Materials and methods

### Sample collection

#### Initial method development

For initial method development commercially available whole horse blood was used (Fisher Scientific UK Ltd., Loughborough UK) as well as anonymized adult human venous blood. From these whole EDTA blood samples, 50 μl aliquots were then immediately spotted onto standard untreated filter paper cards (Ahlstrom 226, ID Biological Systems; as used in national UK newborn bloodspot screening) and air dried for up to 4 h. From the same blood samples, 2 ml aliquots were additionally frozen as whole blood and 2 ml aliquots were processed to plasma, allowing us to determine if lipid profiles from these different sample types were comparable. All samples were then stored at or below −20 °C.

#### QC material

Dried blood spot quality control (QC) material was used in all experiments. QC1 spots were made from mixed anonymized adult human venous blood; QC2 spots from commercially obtained horse blood.

#### Infant dried blood spot samples

Infant samples were collected as part of CBGS (Ong et al. 2009), using heel prick blood sampling and dried blood spots were then stored at −20 °C. The CBGS was approved by the Cambridge local research ethics committee, UK and all mothers gave informed written consent. Dried blood spots were obtained from healthy infants at age 3 months (*n* = 40) and 12 months (*n* = 36), all exclusively breast-fed until at least 3 months of age, average growth characteristics are in electronic supplementary material, Table S1. These samples were collected over a 6 year period with the oldest samples collected in March 2004. The samples were balanced for sex and birth weight (details in supplementary data).

For all samples a round disc with diameter of 3.2 mm was punched out and used for analysis. When the text refers to dried blood spot samples, it means 3.2 mm diameter discs punched out from larger blood spots.

### Chemicals

All solvents of LC–MS grade or better were ordered from Sigma Aldrich (Gillingham, UK). All internal standards were obtained from Avanti Polar Lipids (through Instruchem, Delfzijn, NL) with the exception of undecanoic acid and trilaurin (Sigma Aldrich, Gillingham, UK).

### Sample extraction

After initial method development we standardized the sample extraction to the following protocol. An automated method for the extraction of lipids was developed using an Anachem Flexus automated liquid handler (Anachem, Milton Keynes, UK). A total of 80 dried blood spot samples, 4 blanks, 8 QC1 and 4 QC2, all as 3.2 mm diameter discs in 1.2 ml Cryovials, were placed in the wells of a glass coated 2.4 ml deep well plate (Plate+™, Esslab, Hadleigh, UK). Firstly, 100 μl of MilliQ H_2_O was added to each of the wells. Then 250 μl of MeOH was added containing six internal standards (0.6 μM 1,2-di-*o*-octadecyl-sn-glycero-3-phosphocHoline, 1.2 μM 1,2-di-*O*-phytanyl-sn-glycero-3-phosphoethanolamine, 0.6 μM C8-ceramide, 0.6 μM *N*-heptadecanoyl-d-erythro-sphingosylphosporylcholine, 6.2 μM undecanoic acid, 0.6 μM trilaurin), followed by 500 μl of methyl *tert*-butyl ether (MTBE). The plates were then sealed (using Corning aluminium microplate sealing tape Sigma Aldrich Company, UK) and shaken for 10 min at 600 rpm, after which the plate was transferred to a centrifuge and spun for 10 min at 6,000 rpm. Each well in the resulting plate had two layers, with an aqueous layer at the bottom and an organic layer on top.

A 96 head micro-dispenser (Hydra Matrix, Thermo Fisher Ltd., Hemel Hampstead, UK) was used to transfer 200 μl of the organic layer to a glass coated 240 μl low well plate (Plate+™, Esslab, Hadleigh, UK). The plate was transferred to a Genevac EZ-2 evaporator (Genevac Ltd., Ipswich, UK) and dried down. The samples were reconstituted in 25 μl of MTBE and with 90 μl of MS-mix [7.5 mM NH_4_Ac IPA:MeOH (2:1)] using a Hydra Matrix, after which the plate was sealed and stored at −20 °C until analysis.

### Mass spectrometry

#### Direct infusion high resolution MS (DIHRMS)

All samples were infused into an Exactive Orbitrap (Thermo, Hemel Hampstead, UK), using a Triversa Nanomate (Advion, Ithaca US). The Nanomate infusion mandrel was used to pierce the seal of each well before analysis, after which, with a fresh tip, 5 μl of sample was aspirated, followed by an air gap (1.5 μl). The tip was pressed against a fresh nozzle and the sample was dispensed using 0.2 psi nitrogen pressure. Ionization was achieved with a 1.2 kV voltage. The Exactive started acquiring data 20 s after sample aspiration began. After 72 s of acquisition in positive mode the Nanomate and the Exactive switched over to negative mode, decreasing the voltage to −1.5 kV. The spray was maintained for another 66 s, after which the analysis was stopped and the tip discarded, before the analysis of the next sample began. Throughout the analysis the sample plate was kept at 15 °C. Samples were run in row order.

#### LC–MS

A selected subset of samples was analysed using a LTQ-Velos Orbitrap Elite coupled to a Surveyor (both Thermo, Hemel Hampstead, UK) LC system for lipid identification. Ionization was achieved using ion max electrospray source (voltage 4.0 Kv, capillary temperature 275 °C, sheath gas 20 arbitrary (arb) units, aux gas 5 arb, sweep gas 10 arb). For each sample, 10 μl was injected onto a Hypersil Gold C18 (50 × 2.1 mm, 1.9 μm particle size) column and lipids were eluted with a gradient of MeOH/H_2_O and MeOH/IPA (Bird et al. 2011). The LTQ-Orbitrap Velos was controlled either manually or set-up using the data-dependent precursor selection. Generally the selected masses were isolated with a 1.5 *m*/*z* width in the linear ion trap and then fragmented using either linear ion trap with 35 % relative collision energy or in the higher-energy collision-induced dissociation collision cell, with a range of collision energies from 5 to 75 % relative collision energy. All spectra were recorded in the Orbitrap set at 100,000 resolution.

### Data and statistics

The raw data were converted to .mzXML (MS convert), parsed to R and 50 spectra averaged per mode using XCMS. Further processing was conducted using Peakpicker v2.0, an in-house R script for peak picking, deisotoping and annotating DIMS data sets. Univariate analysis was performed in Prism 6.01 (GraphPad Software, Inc.). All significance thresholds were set using Bonferroni multi-comparison correction. Multivariate analysis was performed in SIMCA (v13.0) (Umetrics). When replicate samples are described in the manuscript it means that separate aliquots of the same sample, or separate 3.2 mm disc of the same spot, were analysed independently.

### Lipid identification

All lipids mentioned in the text were preliminary identified based on accurate mass and subsequently based on accurate mass MS^2^ spectra and retention time from the LC–MS experiment. The identifications, with ChEBI IDs where possible are summarized in electronic supplementary material, Table S2.

## Results and discussion

We have adapted a lipidomic method (Graessler et al. 2009), based on work with human plasma, to assess the suitability of dried blood spots for lipid profiling, and determined the extent of lipid degradation and oxidation during this sampling process. The use of glass-coated well plates was essential to minimize the introduction of contaminants, although non-glass pipette tips still were a possible source of contamination, we did not find that the levels impaired the reproducibility of the lipids of interest (see example spectra in Fig. S1 of electronic supplementary material). We aimed to determine if the dried blood spot samples yielded data of sufficient signal and robustness such that it was comparable to that from plasma. Our novel method only required one 3.2 mm diameter disc, containing 2.5–5 μl of blood, punched from a dried blood spot obtained by heel prick capillary blood sampling. This approach substantially reduced the sample volume required compared to alternative open profiling methods (Dénes et al. 2012). Signal intensities corresponding to specific lipids (see Table S1 of electronic supplementary material) were extracted and summed. The intensity of each lipid species was expressed relative to the summed intensities of selected signals, in the same way as fatty acid profiling by GC is often expressed (Wang et al. 2013). This relative quantitation is therefore independent of the blood volume in the spot and hematocrit level. For the method development and validation, oxidized lipids were also included to the list of target lipids. The purpose of the internal standards added was primarily to check mass accuracy and post run calibration. Our method does not provide absolute concentrations but only relative levels as commonly used in other lipidomics approaches (Graessler et al. 2009; Rhee et al. 2011).

The samples were extracted and processed using an automated liquid handler. A plate containing 80 analytical samples and 16 QC and blank samples could be processed in under 2 h. Lipid extracts were directly infused into a HRMS using chip based nanospray. The analysis time was ca. 2.5 min per sample, which is over 10 times faster than previously reported methods for the profiling of lipids (or fatty acids) from infant blood samples (Escolano-Margarit et al. 2013; Nikkila et al. 2008) and on par with other high-throughput methods for lipid profiling and suitable for the profiling of large sample sets of large epidemiological cohorts.

Dried blood spots are a convenient sample format to store over long time periods and different cohorts have done so. A fast and robust lipid profiling method would therefore provide the opportunity to study lipid metabolism in large epidemiological cohorts. However, some lipids are known to be prone to oxidation and hydrolysis, and it has never been documented how storage time affects the recovery of lipids from dried blood spot samples. It was therefore important to validate the method and determine lipid profile variation due to blood spotting, drying and prolonged storage.

**Fig. 1:**
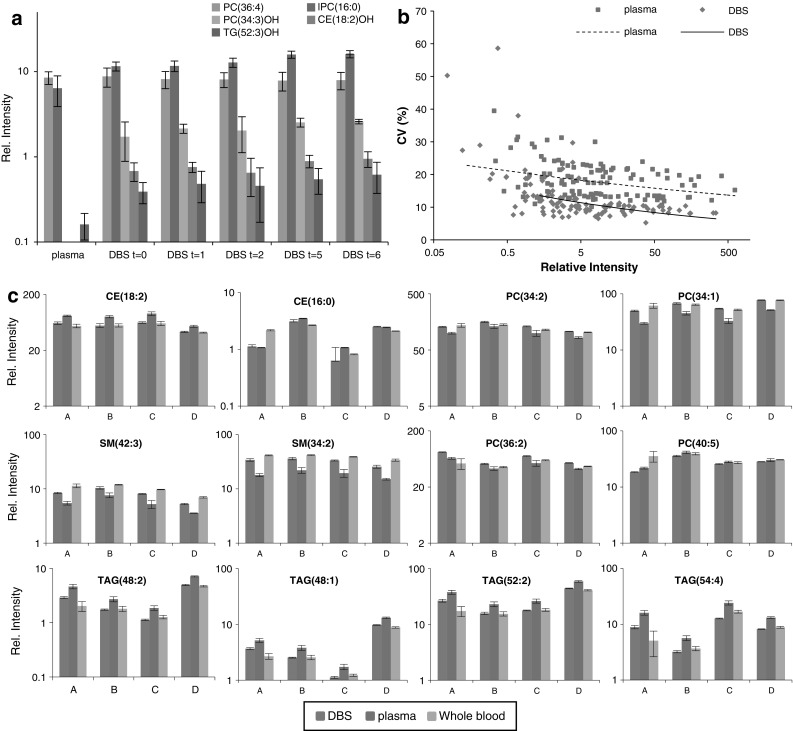
The comparison of lipid profiles from dried blood spots (DBS) versus profiles obtained from plasma or whole blood. Samples were collected from four different volunteers. For each of these, the three sample types (DBS, plasma, Whole blood) were processed independently: **a** representatives of different lipid classes in plasma, DBS and during accelerated aging of DBS (*t* = days at 40 °C), **b** the correlation between the relative intensity of lipids and the precision (expressed as coefficient of variance) for DBS and plasma (*n* = 16), and **c** the relative intensities of different lipids from DBS, plasma and whole blood for four different adults (*A*–*D*), showing comparable inter individual differences across the three sample types (*n* = 4). All *error bars* are standard deviations

We found that spotting and drying had a significant effect on the lipid profile, resulting in multiple oxidized lipids and products of hydrolysis (Fig. 1a). Many different lipid species were prone to oxidation. In principle all lipids containing polyunsaturated fatty acids could be oxidized. We found oxidized cholesteryl esters, triglycerides, phosphatidylcholines, sphingomyelins and phosphoethanolamines (Fig. 1a). However, the oxidized products of these lipids are usually not detected in human plasma or serum samples by this methodology. Further analysis (see Fig. S2 of electronic supplementary material) by LC–MS showed that the oxidized lipids shifted in retention time and HRMS^2^ data showed the addition of oxygen to the fatty acids for instance for CE(18:2)OH (Hutchins et al. 2011). Therefore we rejected these signals from further data analysis. Although the oxidized lipids were clearly detectable, they are the products of only around 5 % or less of the original lipids (this was manually checked for the main lipid species including cholesteryl esters, phosphocholines, sphingomyelins and triglycerides), therefore having a very limited effect on the overall level of the native lipid profile. This was confirmed by LC–MS demonstrating that the signal of oxidized lipids was only a few percent of that of the substrate (see Fig. S2 of electronic supplementary material). For instance, PC(40:6) (*m*/*z* 834.600) showed a decrease of less than 2 % and PC(36:4) less than 3 % (see Fig. 1a) during our accelerated aging experiment.

Importantly, the precision of the dried blood spot based lipid profiling was not different to that based on whole blood samples for the known identified lipid species (Fig. 1b), such as triglycerides, sphingomyelins and phosphatidylcholines, encompassing many lipids associated with risk for developing type II diabetes (Floegel et al. 2012; Rhee et al. 2011). Generally the coefficient of variance (CV) was below 20 %, except for those lipids with concentrations around the limit of detection, which was around a 1,000-fold lower level than the most dominant lipids (Fig. 1b). The lipid profiles of dried blood spots revealed the same pattern of inter-subject differences as plasma or whole blood samples (Figs. 1c, S3 of electronic supplementary material) with comparable or better precision (Fig. 1b). This showed that any degradation processes did not introduce extra variability in the major lipid species. The average CV for the lipids measured in the dried blood spot samples was 8.7 % while it was 10.6 for the plasma samples, which was a significant difference (*p* < 0.05). The reason that dried blood spots yielded more precise measurements could be that for each sample, oxidative and other degrading processes are stopped once the spots are fully dried, while in plasma samples the enzymatic and chemical processes can occur once the sample is defrosted, and therefore more difficult to control.

We also wanted to simulate longer term storage, which we did using an accelerated aging experiment; dried blood spots were stored at 40 °C for up to a week. This did not lead to any further significant changes, suggesting that the major changes observed take place during initial blood spotting and drying, with no further significant effect on the lipid levels with prolonged storage (see Fig. 1a). Since most lipid profiling studies based on plasma or serum use venepuncture (Floegel et al. 2012; Rhee et al. 2011), we determined if there was a difference in whole blood collected by venepuncture or collected from the capillary bed (heel prick or finger prick). We found that there were no significant relative changes in the lipid profile between dried blood spots of venous versus blood of the arterial bed (see Fig. S4 in electronic supplementary material).

When we were confident that the dried blood spots were able to provide a representative and robust lipid profile, we applied our new method to study the lipid profiles of healthy infants using a subset of samples from the CBGS (Ong et al. 2009). Of note, we had no difficulty in obtaining lipid profiles from the samples and the sample age did not show a significant correlation with the total lipid extracted or with any individual lipid classes, in accordance with our validation results.

**Fig. 2:**
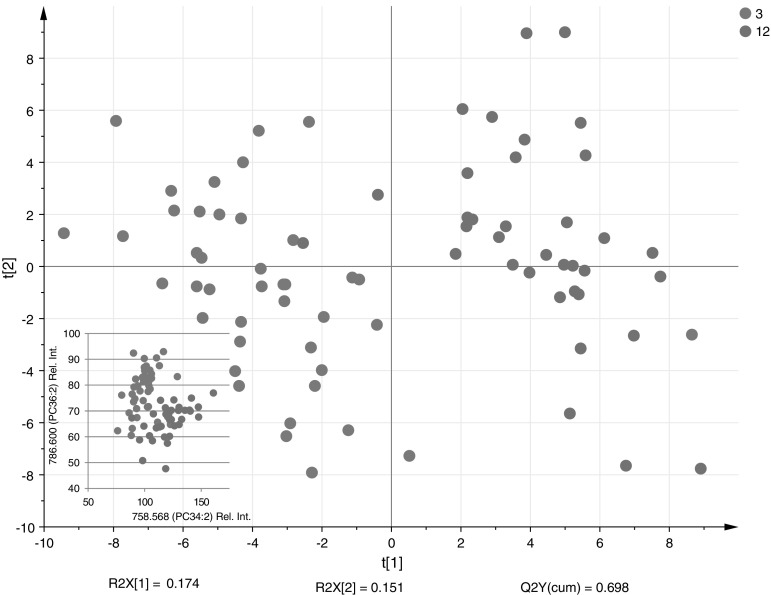
PLS-DA plot showing the separation of the lipid profiles of 3 and 12 month old infant (*n* = 40 and 37) from the Cambridge Baby Growth Study. *Inset* graph based on the same samples expressing the relative levels of PC(34:2) (*m*/*z* 758.568) and PC(36:2) (*m*/*z* 786.600) resulting in the separation of the two age classes

There were striking differences between the lipid profile of 3 month old infants and those at 12 months of age. Both showed differences to the lipid profiles of dried blood spots of adults. The differences were across different lipid classes and overall separation is clear in the partial least square discriminant analysis (PLS-DA) in Fig. 2. This model passed cross validation with a *Q* ^2^ of 0.698, which showed that the model was robust. In the PLS-DA model one of the samples from a 3 month old infant was misclassified within the 12 month group, but this was the only sample. For all the differentiating lipids this sample showed values characteristic for the 12 month group.

The major differences between the two age groups were the relative amount of palmitate versus stearate in the phosphatidylcholines fraction. A previous study showed a similar difference when comparing the fatty acid profile of plasma phospholipids between mothers and preterm infants (Sabel et al. 2009). The differences we found were dramatic over the first year of life. PC(34:2), mainly containing palmitate at sn-1 position, was increased in the 12 month old infants and PC(36:2), mainly containing stearate at sn-1 position, was increased in the 3 month old infants. The ratio between the two phosphatidylcholines was an almost perfect predictor for the two age groups (*p* = 6.9e^−22^), and at 12 months this was already much closer to that observed in adults (see inset of Fig. 2). It was not possible to build a robust model based on sex, although there was a trend in the data towards increased triglycerides in boys, particularly at 12 months of age, though not significant.

**Fig. 3:**
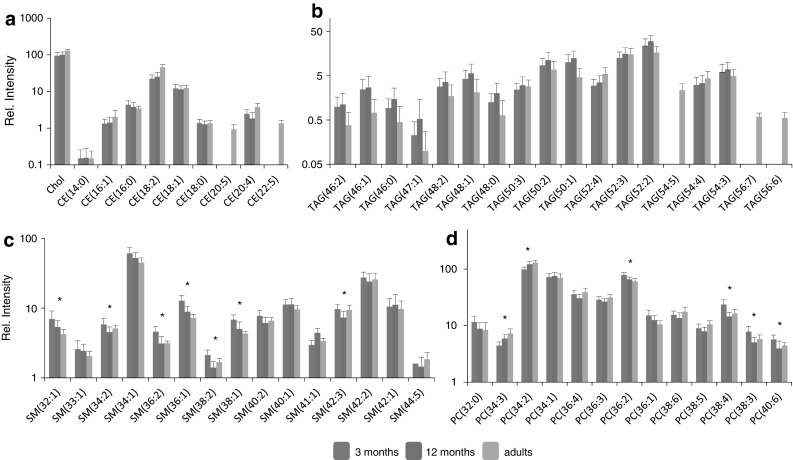
The most abundant lipids from dried blood spot lipid profiles of infants at 3 and 12 months of age, showing average values (±standard deviation, *n* = 76) in comparison to the levels found in dried blood spots of healthy adults, *significant difference between 3 and 12 months (*p* < 0.0003, based on a Bonferroni correction for multi comparison of 137 lipid signals). **a** Cholesteryl esters (measured as the ${\text{NH}}_{4}^{ + }$ adducts), **b** triglycerides (measured as the ${\text{NH}}_{4}^{ + }$ adducts), **c** sphingomyelins, and **d** phosphatidylcholine lipids (full list of lipids and ions selected for the profile can be found in electronic supplementary material: Tables S1, S2)

The lipid profiles revealed that infants predominately transport long-chain poly-unsaturated fatty acids as phosphatidylcholines (see Fig. 3), rather than in other lipid classes such as cholesteryl esters and triglycerides. Unlike in adults, where cholesteryl esters of n-3 polyunsaturated fatty acids and triglycerides can easily be detected, the levels in the infant samples were below the level of detection (see Fig. 3a, b). In adults, long-chain poly-unsaturated fatty acids like DHA and EPA form around 0.4 % of the total fatty acid content of triglycerides (Hodson et al. 2008), whilst in contrast for young infants the only report available (based on 12 infants age 6 months) showed levels less than 0.2 % of these fatty acids in triglycerides (Decsi and Koletzko 2000). Our results confirm these observations but on a larger groups of infants and with a much faster and robust profiling technique. The triglycerides of infants contained relatively more saturated fatty acids like palmitate and myristate (see Fig. 3b). We hypothesize that the large overall differences in the circulating unsaturated fatty acid structure might be aimed to traffic these to the developing brain, to supply it with sufficient polyunsaturated fatty acids. In adults polyunsaturated fatty acids are, with other fatty acids, part of triglyceride and cholesteryl ester fraction, and therefore in the VLDL and LDL component of blood, while in young infants the polyunsaturated fatty acid are mainly part of phosphatidylcholine fraction and therefore more abundant in the HDL components than in the larger lipoprotein particles. Although there is limited knowledge on lipid transport to the brain it has been shown that HDL can be transported across the blood–brain barrier (Balazs et al. 2004).

The other striking difference was the level of sphingomyelins (see Fig. 3c) which was 25–50 % higher in the 3 month old, compared to adults and still 10 % higher in the samples of 12 month old [only SM(41:1) was significantly higher in the 12 month old infants]. Sphingomyelins are the most abundant phospholipids in human breast milk (Jensen et al. 1978) and the higher concentration in the 3 month old infants might be due to the metabolism of the breast milk fats (Nilsson and Duan 2006). Previous studies suggested that lipid metabolism is rapidly changing within childhood (Nikkila et al. 2008) but to our knowledge, it has not been previously documented that these differences are significant within the first year of life.

## Concluding remarks

We believe that lipid profiles of dried blood spots are an important new tool to study the metabolism of infants, and have successfully shown novel methodology to do this. The developed method is fast and requires only a 3.2 mm diameter disc of a dried blood spot. It is therefore compatible with simultaneous biochemical or genetics analyses of the same sample. Although the spotting and drying of the blood has a distinct effect on the lipid profile it is still possible to obtain information on global lipid metabolism changes, comparable to lipid profiles obtained from plasma collected through venepuncture. This now gives us the unique opportunity to explore infancy lipidomics and relationships with later anthropometric and metabolic outcomes in large epidemiological study cohorts using samples already gathered, for example during the newborn bloodspot test. We hypothesize this will lead to better understanding into the early biological mechanisms of insulin resistance and related metabolic diseases.

## Electronic supplementary material

Below is the link to the electronic supplementary material.

Supplementary material 1 (DOCX 864 kb)

## References

[CR1] Abu-Rabie P, Spooner N (2009). Direct quantitative bioanalysis of drugs in dried blood spot samples using a thin-layer chromatography mass spectrometer interface. Analytical Chemistry.

[CR2] Balazs Z, Panzenboeck U, Hammer A, Sovic A, Quehenberger O, Malle E, Sattler W (2004). Uptake and transport of high-density lipoprotein (HDL) and HDL-associated alpha-tocopherol by an in vitro blood–brain barrier model. Journal of Neurochemistry.

[CR3] Barker DJ, Bull AR, Osmond C, Simmonds SJ (1990). Fetal and placental size and risk of hypertension in adult life. British Medical Journal.

[CR4] Bird SS, Marur VR, Sniatynski MJ, Greenberg HK, Kristal BS (2011). Serum lipidomics profiling using LC–MS and high-energy collisional dissociation fragmentation: Focus on triglyceride detection and characterization. Analytical Chemistry.

[CR5] Chace DH, Millington DS, Terada N, Kahler SG, Roe CR, Hofman LF (1993). Rapid diagnosis of phenylketonuria by quantitative analysis for phenylalanine and tyrosine in neonatal blood spots by tandem mass spectrometry. Clinical Chemistry.

[CR6] Decsi T, Koletzko B (2000). Effects of protein–energy malnutrition and human immunodeficiency virus-1 infection on essential fatty acid metabolism in children. Nutrition.

[CR7] Dénes J, Szabo E, Robinette SL, Szatmari I, Szonyi L, Kreuder JG, Rauterberg EW, Takats Z (2012). Metabonomics of newborn screening dried blood spot samples: A novel approach in the screening and diagnostics of inborn errors of metabolism. Analytical Chemistry.

[CR8] Escolano-Margarit MV, Campoy C, Ramirez-Tortosa MC, Demmelmair H, Miranda MT, Gil A, Decsi T, Koletzko BV (2013). Effects of fish oil supplementation on the fatty acid profile in erythrocyte membrane and plasma phospholipids of pregnant women and their offspring: A randomised controlled trial. The British Journal of Nutrition.

[CR9] Floegel A, Stefan N, Yu Z, Mühlenbruch K, Drogan D, Joost H-G, Fritsche A, Häring H-U, Hrabe de Angelis M, Peters A, Roden M, Prehn C (2012). Identification of serum metabolites associated with risk of type 2 diabetes using a targeted metabolomic approach. Diabetes.

[CR10] Graessler J, Schwudke D, Schwarz PE, Herzog R, Shevchenko A, Bornstein SR (2009). Top-down lipidomics reveals ether lipid deficiency in blood plasma of hypertensive patients. PLoS ONE.

[CR11] Harder T, Rodekamp E, Schellong K, Dudenhausen JW, Plagemann A (2007). Birth weight and subsequent risk of type 2 diabetes: A meta-analysis. American Journal of Epidemiology.

[CR12] Hattersley AT, Tooke JE (1999). The fetal insulin hypothesis: An alternative explanation of the association of low birthweight with diabetes and vascular disease. Lancet.

[CR13] Hodson L, Skeaff CM, Fielding BA (2008). Fatty acid composition of adipose tissue and blood in humans and its use as a biomarker of dietary intake. Progress in Lipid Research.

[CR14] Hutchins PM, Moore EE, Murphy RC (2011). Electrospray tandem mass spectrometry reveals extensive and non-specific oxidation of cholesterol esters in human peripheral vascular lesions. Journal of Lipid Research.

[CR15] Jensen RG, Hagerty MM, McMahon KE (1978). Lipids of human milk and infant formulas: A review. American Journal of Clinical Nutrition.

[CR16] Kuipers RS, Luxwolda MF, Sango WS, Kwesigabo G, Velzing-Aarts FV, Dijck-Brouwer DA, Muskiet FA (2011). Postpartum changes in maternal and infant erythrocyte fatty acids are likely to be driven by restoring insulin sensitivity and DHA status. Medical Hypotheses.

[CR17] Nikkila J, Sysi-Aho M, Ermolov A, Seppanen-Laakso T, Simell O, Kaski S, Oresic M (2008). Gender-dependent progression of systemic metabolic states in early childhood. Molecular Systems Biology.

[CR18] Nilsson A, Duan RD (2006). Absorption and lipoprotein transport of sphingomyelin. Journal of Lipid Research.

[CR19] Ong KK, Langkamp M, Ranke MB, Whitehead K, Hughes IA, Acerini CL, Dunger DB (2009). Insulin-like growth factor I concentrations in infancy predict differential gains in body length and adiposity: The Cambridge Baby Growth Study. The American Journal of Clinical Nutrition.

[CR20] Pandor A, Eastham J, Beverley C, Chilcott J, Paisley S (2004). Clinical effectiveness and cost-effectiveness of neonatal screening for inborn errors of metabolism using tandem mass spectrometry: A systematic review. Health Technology Assessment.

[CR21] Rhee E, Cheng S, Larson M, Walford G, Lewis G, McCabe E, Yang E, Farrell L, Fox C, O’Donnell C, Carr S, Vasan R (2011). Lipid profiling identifies a triacylglycerol signature of insulin resistance and improves diabetes prediction in humans. The Journal of Clinical Investigation.

[CR22] Sabel KG, Lundqvist-Persson C, Bona E, Petzold M, Strandvik B (2009). Fatty acid patterns early after premature birth, simultaneously analysed in mothers’ food, breast milk and serum phospholipids of mothers and infants. Lipids in Health and Disease.

[CR23] Wang LY, Summerhill K, Rodriguez-Canas C, Mather I, Patel P, Eiden M, Young S, Forouhi NG, Koulman A (2013). Development and validation of a robust automated analysis of plasma phospholipid fatty acids for metabolic phenotyping of large epidemiological studies. Genome Medicine.

