# Supplementary material for: The development and validation of a fast and robust dried blood spot based lipid profiling method to study infant metabolism
Source: Metabolomics. 2014 Feb 11;10(5):1018–25. doi: 10.1007/s11306-014-0628-z (PMC4145199; doi:10.1007/s11306-014-0628-z)
Supplement: Supplementary file 1 — Supplementary material 1 (DOCX 864 kb) [file 11306_2014_628_MOESM1_ESM.docx]

**Electronic supplementary material**

The development and validation of a fast and robust dried blood spot based lipid profiling method to study infant metabolism

Table S1 Birth and growth characteristics of infants divided by sex and age when samples were taken and the relative lipid levels (mean (±SD)).

|  | |  | Male | Female | Male | Female |
| --- | --- | --- | --- | --- | --- | --- |
|  | |  | 3 months | | 12 months | |
|  | |  | n=20 | n=20 | n=17 | n=19 |
| Gestational Age | | (weeks) | 40.39(1.12) | 39.86(1.07) | 40.26(1.02) | 40.38(1.05) |
| Birth weight | | (kg) | 3.55(0.48) | 3.41(0.36) | 3.57(0.36) | 3.35(0.35) |
| Weight at 3 m | | (kg) | 6.43(0.66) | 5.76(0.43) | 6.51(0.68) | 5.65(0.51) |
| Weight at 12 m | | (kg) | 10.14(0.81) | 9.48(0.51) | 10(0.73) | 9.18(0.66) |
|  | |  |  |  |  |  |
| Lipid | m/z | |  |  |  |  |
| Chol | 369.351 | | 11.41(2.43) | 10.73(3.04) | 11.64(2.52) | 12(2.46) |
| CE(16:0) | 642.618 | | 0.55(0.14) | 0.47(0.15) | 0.43(0.12) | 0.47(0.16) |
| CE(18:2) | 666.618 | | 2.86(0.71) | 2.38(0.72) | 2.93(1.11) | 2.99(0.81) |
| CE(18:1) | 668.633 | | 1.52(0.42) | 1.33(0.45) | 1.29(0.33) | 1.4(0.38) |
| CE(20:4) | 690.618 | | 0.32(0.1) | 0.26(0.09) | 0.21(0.11) | 0.23(0.1) |
| PC(30:0) | 706.538 | | 0.26(0.06) | 0.26(0.09) | 0.23(0.05) | 0.19(0.06) |
| PC(32:1) | 732.551 | | 0.51(0.16) | 0.52(0.12) | 0.6(0.19) | 0.54(0.16) |
| PC(32:0) | 734.569 | | 1.3(0.32) | 1.4(0.36) | 1.06(0.26) | 1.01(0.27)* |
| PC(34:3) | 756.552 | | 0.51(0.08) | 0.53(0.08) | 0.7(0.1) | 0.69(0.13)* |
| PC(34:2) | 758.568 | | 11.51(0.89) | 11.58(1.35) | 14.35(1.2) | 14.29(2)* |
| PC(34:1) | 760.592 | | 8.2(1.12) | 8.66(1.13) | 8.99(0.96) | 8.79(1.28) |
| PC(35:2) | 772.579 | | 0.25(0.05) | 0.23(0.05) | 0.32(0.09) | 0.31(0.08)* |
| PC(36:4) | 782.568 | | 4.16(0.49) | 4.21(0.87) | 3.6(0.5) | 3.61(0.61) |
| PC(36:3) | 784.584 | | 3.34(0.39) | 3.35(0.43) | 3.15(0.34) | 3.1(0.34) |
| PC(36:2) | 786.601 | | 8.98(1) | 9.43(0.79) | 7.88(0.65) | 7.65(0.77)* |
| PC(36:1) | 788.616 | | 1.63(0.42) | 1.9(0.36) | 1.54(0.3) | 1.41(0.21) |
| PC(37:4)^b^ | 796.573 | | 0.07(0.02) | 0.07(0.03) | 0.07(0.03) | 0.07(0.03) |
| PC(38:6) | 806.564 | | 1.83(0.27) | 1.8(0.32) | 1.65(0.4) | 1.58(0.38) |
| PC(38:5) | 808.584 | | 1.03(0.19) | 1.07(0.21) | 0.92(0.18) | 0.94(0.17) |
| PC(38:4) | 810.606 | | 2.69(0.43) | 2.82(0.68) | 1.73(0.28) | 1.69(0.38)* |
| PC(38:2) | 812.616 | | 0.9(0.15) | 0.92(0.27) | 0.63(0.14) | 0.56(0.12)* |
| PC(40:6) | 834.599 | | 0.68(0.13) | 0.66(0.12) | 0.5(0.13) | 0.44(0.19)* |
| PC(40:5) | 836.618 | | 0.13(0.05) | 0.13(0.07) | 0.13(0.05) | 0.11(0.04) |
| SM(34:2) | 701.555 | | 0.69(0.15) | 0.67(0.14) | 0.51(0.07) | 0.55(0.11)* |
| SM(34:1) | 703.572 | | 6.97(1.3) | 7.2(1.63) | 6.14(1.09) | 6.12(1.13) |
| SM(36:2) | 729.590 | | 0.54(0.11) | 0.53(0.1) | 0.35(0.09) | 0.38(0.1)* |
| SM(36:1) | 731.605 | | 1.46(0.25) | 1.52(0.3) | 1.05(0.17) | 1.03(0.22)* |
| SM(38:1) | 759.637 | | 0.77(0.11) | 0.81(0.15) | 0.64(0.18) | 0.55(0.1)* |
| SM(40:2) | 785.654 | | 0.87(0.14) | 0.93(0.18) | 0.74(0.15) | 0.69(0.13)* |
| SM(40:1) | 787.670 | | 1.28(0.21) | 1.34(0.22) | 1.39(0.31) | 1.27(0.26) |
| SM(41:1) | 801.684 | | 0.35(0.05) | 0.34(0.05) | 0.54(0.07) | 0.5(0.08)* |
| SM(42:3) | 811.668 | | 1.1(0.15) | 1.16(0.22) | 0.85(0.19) | 0.86(0.2)* |
| SM(42:2) | 813.685 | | 3.08(0.47) | 3.31(0.68) | 2.86(0.86) | 2.78(0.69) |
| SM(42:1) | 815.703 | | 1.18(0.38) | 1.26(0.38) | 1.39(0.55) | 1.24(0.46) |
| PE(34:1) | 718.538 | | 0.26(0.06) | 0.26(0.09) | 0.23(0.05) | 0.19(0.06) |
| PE(36:4)^c^ | 740.521 | | 0.01(0.01) | 0.01(0.01) | 0.02(0.01) | 0.02(0.01) |
| PE(36:3)^c^ | 742.538 | | 0.05(0.02) | 0.05(0.02) | 0.07(0.02) | 0.06(0.02) |
| PE(36:2)^c^ | 744.553 | | 0.1(0.02) | 0.1(0.02) | 0.17(0.04) | 0.15(0.04)* |
| PE(38:4) | 768.549 | | 0.08(0.02) | 0.07(0.03) | 0.09(0.03) | 0.07(0.03) |
| PE(40:6) | 792.534 | | 0.07(0.02) | 0.07(0.03) | 0.07(0.03) | 0.07(0.03) |
| TAG(46:2) | 792.707 | | 0.14(0.09) | 0.1(0.05) | 0.15(0.1) | 0.12(0.11) |
| TAG(46:1) | 794.723 | | 0.34(0.24) | 0.23(0.1) | 0.36(0.22) | 0.28(0.27) |
| TAG(47:1) | 808.739 | | 0.03(0.02) | 0.02(0.03) | 0.09(0.15) | 0.04(0.04) |
| TAG(48:2) | 820.739 | | 0.39(0.21) | 0.28(0.11) | 0.46(0.25) | 0.39(0.32) |
| TAG(48:1) | 822.758 | | 0.58(0.32) | 0.41(0.17) | 0.73(0.35) | 0.61(0.45) |
| TAG(48:0) | 824.771 | | 0.17(0.09) | 0.13(0.07) | 0.25(0.13) | 0.22(0.17) |
| TAG(50:3) | 846.754 | | 0.32(0.12) | 0.25(0.08) | 0.37(0.19) | 0.35(0.2) |
| TAG(50:2) | 848.769 | | 1.13(0.45) | 0.88(0.34) | 1.35(0.61) | 1.32(0.67) |
| TAG(50:1) | 850.786 | | 1.3(0.55) | 1.06(0.53) | 1.45(0.56) | 1.5(0.7) |
| TAG(51:2) | 862.786 | | 0.11(0.04) | 0.08(0.04) | 0.17(0.08) | 0.15(0.09) |
| TAG(52:4) | 872.769 | | 0.36(0.12) | 0.33(0.15) | 0.4(0.18) | 0.43(0.18) |
| TAG(52:3) | 874.785 | | 1.56(0.48) | 1.37(0.57) | 1.72(0.58) | 1.94(0.76) |
| TAG(52:2) | 876.801 | | 2.96(1.1) | 2.54(1.27) | 3.22(0.98) | 3.68(1.44) |
| TAG(53:2) | 890.817 | | 0.07(0.03) | 0.05(0.03) | 0.08(0.03) | 0.09(0.05) |
| TAG(54:4) | 900.801 | | 0.36(0.14) | 0.36(0.2) | 0.35(0.15) | 0.45(0.2) |
| TAG(54:3) | 902.817 | | 0.73(0.34) | 0.67(0.34) | 0.68(0.23) | 0.9(0.45) |

^a^: PC(33:2) and PE(36:3) have the same exact m/z as and subsequent analysis by LCMS showed that both lipids contribute significantly to this signal. b: PC(33:1) and PE(36:2) have the same exact m/z as and subsequent analysis by LCMS showed that both lipids contribute significantly to this signal. ^c^: PC(37:4) and PE(40:4) have the same exact m/z as and subsequent analysis by LCMS showed that both lipids contribute significantly to this signal.

Table S-2 Identification of lipids detected in infant dried blood spot samples.

|  |  |  |  |  |  |  |
| --- | --- | --- | --- | --- | --- | --- |
| Lipid | ChEBI^a^ | m/z | Rt | MS^2^ Spectrum |  | Most abundant lipid species |
| Chol (M-H_2_O+H^+^) | 16113 | 369.351 | 23.2 | 109.101(36); 135.117(53); 147.117(72); 149.132(32); 161.132(69); 175.148(30); 243.211(31); 259.242(30) | |  |
| CE(16:0) (M+NH_4_^+^) | **3663** | 642.618 | 45.9 | MS2 647.57: 279.230(100), 369.315(15) | |  |
| CE(18:2) (M+NH_4_^+^) | **41509** | 666.618 | 44.2 | MS2 671.57: 303.230(100); 369.315(12) | |  |
| CE(18:1) (M+NH_4_^+^) | **46898** | 668.633 | 45.8 | MS2 673.57: 305.245(100); 369.315(13) | |  |
| CE(20:4) (M+NH_4_^+^) |  | 690.618 | 43.4 | MS2 695.57: 327.230(100); 369.315(10) | |  |
| PC(30:0) (M+H^+^) | **65303** | 706.538 | 27.2 | 125.000, 184.073, 478.329; 496.335 | | PC(16:0/14:0) |
| PC(32:1) (M+H^+^) | **66849** | 732.551 | 26.8 | 125.000 (5); 184.073(100); 476.313 (<1); 478.330(<1); 496.339(<1); 549.490(<1) | | PC(16:0/16:1) |
| PC(32:0) (M+H^+^) | **65302** | 734.569 | 31.2 | 125.000(5); 184.073; 478.330(<1); 496.339(<1); 551.504(<1) | | PC(16:0/16:0) |
| PC(34:3) (M+H^+^) | **64424** | 756.552 | 24.5 | 125.000(5); 184.073(100); 476.313(<1); 494.325(<1); 502.326(<1); 573.500(<1) | | PC(16:1/18:2) |
| PC(34:2) (M+H^+^) | **64516** | 758.568 | 27.1 | 125.000(5); 184.073(100); 478.330(<1); 496.339(<1); 502.328(<1); 575.503(<1) | | PC(16:0/18:2) |
| PC(34:1) (M+H^+^) | **64517** | 760.592 | 30.4 | 125.000(5); 184.073(100); 478.330(<1); 496.339(<1); 504.348(<1); 577.519(<1) | | PC(16:0/18:1) |
| PC(35:2) (M+H^+^) |  | 772.579 |  |  |  |  |
| PC(36:4) (M+H^+^) | **64520** | 782.568 | 25 | 125.000(5); 184.073(100); 478.330(<1); 496.339(<1); 526.330 (<1) | | PC(16:0/20:4) |
| PC(36:3) (M+H^+^) | **64523** | 784.584 | 27.4 | 125.000(5); 184.073(100); 502.330(<1); 504.346(<1); 522.357(<1) | | PC(18:1/18:2) |
| PC(36:2) (M+H^+^) | **64433** | 786.601 | 30.7 | 125.000(5); 184.073(100); 502.330(<1); 506.364(<1); 524.370(<1); 603.535(<1) | | PC(18:0/18:2) |
| PC(36:1) (M+H^+^) | **66857** | 788.616 | 35.8 | 125.000(5); 184.073(100); 504.343(<1); 506.361(<1); 524(<1).368(<1); 605.551(<1) | | PC(18:0/18:1) |
| PC(37:4)^b^(M+H^+^) | **72427** | 796.573 |  |  |  |  |
| PC(38:6) (M+H^+^) | **64519** | 806.564 | 24.5 | 125.000(5); 184.073(100); 478.33(<1)0; 496.339(<1); 550.330(<1); 623.504(<1) | | PC(16:0/22:6) |
| PC(38:5) (M+H^+^) | **64525** | 808.584 | 26.2 | 125.000(5); 184.073(100); 504.346(<1); 522.357(<1); 526.331(<1) | | PC(18:1/20:4) |
| PC(38:4) (M+H^+^) | **64526** | 810.606 | 29.3 | 125.000(5); 184.073(100); 506.362(<1); 524.370(<1); 526.331(<1) | | PC(18:0/20:4) |
| PC(38:2) (M+H^+^) | **66859** | 812.616 | 32.0 | 125.000(5); 184.073(100); 506.362(<1); 524.370(<1); 528.343(<1) | | PC(18:0/20:3) |
| PC(40:6) (M+H^+^) | **64431** | 834.599 | 28.0 | 125.000(5); 184.073(100); 506.362(<1); 524.370(<1); 550.330(<1) | | PC(18:0/22:6) |
| PC(40:5) (M+H^+^) | **64524** | 836.618 | 29.5 | 125.000(5); 184.073(100); 506.362(<1); 524.370(<1); 552.344(<1) | | PC(18:0/22:5) |
| SM(34:2) (M+H^+^) | **64587** | 701.555 | 22.9 | 125.000(5); 184.073(100); 262.253(<1); 500.485(<1); 683.548(<1) | | SM(d18:2/16:0) |
| SM(34:1) (M+H^+^) | **72514** | 703.572 | 25.7 | 125.000(5); 184.073(100); 264.269(<1); 502.500(<1); 683.565(<1) | | SM(d18:1/16:0) |
| SM(36:2) (M+H^+^) | **72519** | 729.590 | 25.7 | 125.000(5); 184.073(100); 262.253(<1); 528.517(<1); 711.577(<1) | | SM(d18:2/18:0) |
| SM(36:1) (M+H^+^) | **72518** | 731.605 | 29.9 | 125.000(5); 184.073(100); 264.269(<1); 530.531(<1); 713.595(<1) | | SM(d18:1/18:0) |
| SM(38:1) (M+H^+^) | **72523** | 759.637 | 34.3 | 125.000(5); 184.073(100); 264.269(<1) | | SM(18:1/20:0) |
| SM(40:2) (M+H^+^) | **72529** | 785.654 | 34.3 | 125.000(5); 184.073(100); 262.253(<1) | | SM(d18:2/22:0) |
| SM(40:1) (M+H^+^) | **72528** | 787.670 | 39.8 | 125.000(5); 184.073(100); 264.269(<1) | | SM(d18:1/22:0) |
| SM(41:1) (M+H^+^) |  | 801.684 | 43.6 | 125.000(5); 184.073(100); 264.269(<1) | | SM(d18:1/23:0) |
| SM(42:3) (M+H^+^) | **72535** | 811.668 | 33.9 | 125.000(5); 184.073(100); 262.253(<1) | | SM(d18:1/24:3) |
| SM(42:2) (M+H^+^) | **72534** | 813.685 | 38.5 | 125.000(5); 184.073(100); 264.269(<1) | | SM(d18:1/24:2) |
| SM(42:1) (M+H^+^) | **72533** | 815.703 | 42.2 | 125.000(5); 184.073(100); 264.269(<1) | | SM(d18:1/24:1) |
| PE(34:1) (M+H^+^) | **71720** | 718.538 | 21.6 | 313.275(4);577.519(100) | | PE(16:0/18:1) |
| PE(36:4)^c^(M+H^+^) | **71730** | 740.521 | 22.1 | 313.27(8); 599.503(100) | | PE(16:0/20:4) |
| PE(36:3)^c^(M+H^+^) | **71729** | 742.538 | 22.2 | 339.290(18); 601.519(100) | | PE(18:1/18:2) |
| PE(36:2)^c^(M+H^+^) | **71728** | 744.553 | 24.9 | 341.305(10); 603.534(100) | | PE(18:0/18:2) |
| PE(38:4) (M+H^+^) | **71737** | 768.549 | 28.4 | 341.305(25); 627.534(100) | | PE(18:0/20:4) |
| PE(40:6) (M+H^+^) | **71746** | 792.534 |  | 341.305(8); 651.534(100) | | PE(18:0/22:6) |
| TAG(46:2) (M+NH_4_^+^) |  | 792.707 | 38.6 | 493.426 (24); 495.440(12); 519.441(24); 521.456(100); 533.456(6); 535.472(21); 549.488(38) | |  |
| TAG(46:1) (M+NH_4_^+^) |  | 794.723 | 40.4 | 495.440(21); 507.439(14); 509.457(21); 521.456(32); 523.471(100); 535.471(56); 537.487(17); 549.487(33); 551,502(13); 563.5029(16); 577.516(7) | |  |
| TAG(47:1) (M+NH_4_^+^) |  | 808.739 | 41.2 | 509.456(34); 521.456(13); 523.471(17); 535.471(45); 537.487(100); 549.487(47); 551.502(10); 563.503(24), 565.516(6); 577.516(7) | |  |
| TAG(48:2) (M+NH_4_^+^) |  | 820.739 | 40.4 | 521.456(20); 547.472(62); 549.487(100); 575.503(5); 577.516(7) | |  |
| TAG(48:1) (M+NH_4_^+^) |  | 822.758 | 42.1 | 523.471(42), 549.487(100), 551,502(29), 577.519(27) | |  |
| TAG(48:0) (M+NH_4_^+^) |  | 824.771 | 42.1 | 523.471(9); 537.489(5); 551.502(100), 565.519(5); 579.535(7) | |  |
| TAG(50:3) (M+NH_4_^+^) |  | 846.754 | 40.6 | 547.472(43); 549.488(100); 573.488(71); 575.503(77), 601.529(32) | |  |
| TAG(50:2) (M+NH_4_^+^) |  | 848.769 | 42.1 | 549.487(100); 575.503(76); 577.519(54); 603.533(10) | |  |
| TAG(50:1) (M+NH_4_^+^) |  | 850.786 | 43.6 | 549.487(4), 551.503(65), 577.519(100), 579.536(2), 605.550(2) | |  |
| TAG(51:2) (M+NH_4_^+^) |  | 862.786 | 42.8 | 549.487(3); 563.503(100); 565.519(5), 575.503(8), 577.519(34); 589.519(38), 591.535(8), 603.533(30) | |  |
| TAG(52:4) (M+NH_4_^+^) |  | 872.769 | 40.9 | 573.488(30), 575.503(100), 577.519(25), 599.503(96), 601.518(16) | |  |
| TAG(52:3) (M+NH_4_^+^) |  | 874.785 | 42.3 | 575.503(86), 577.519(85), 601.518(100), 603.533(7) | |  |
| TAG(52:2) (M+NH_4_^+^) |  | 876.801 | 43.5 | 577.519(100); 603.534(55) | |  |
| TAG(53:2) (M+NH_4_^+^) |  | 890.817 | 42.0 | 577.518(18); 589(11); 591.534(100); 593.549(4); 603.534(45); 605.550(8); 617.550(19) | |  |
| TAG(54:4) (M+NH_4_^+^) |  | 900.801 | 42.3 | 575.503(2); 577.518(7); 601.519(100); 603.534(55); 605.550(2); 627.535(5) | |  |
| TAG(54:3) (M+NH_4_^+^) |  | 902.817 | 43.5 | 577.519(2); 601.519(13); 603.534(100); 605.550(11); 629.550(3) |  |  |

^a^: CHEBI (database and ontology of Chemical Entities of Biological Interest) ID: further details of the lipids can be found on <http://www.ebi.ac.uk/chebi/>. ^b^: PC(37:4) and PE(40:4) have the same exact m/z as and subsequent analysis by LCMS showed that both lipids contribute significantly to this signal. ^c^: PE(36:4), PE(36:3) and PE(36:2) have the same exact m/z as PC(33:3), PC(33:2) and PC(33:1) respectively ,subsequent analysis by LCMS showed that PE lipids contribute mainly to this signal.


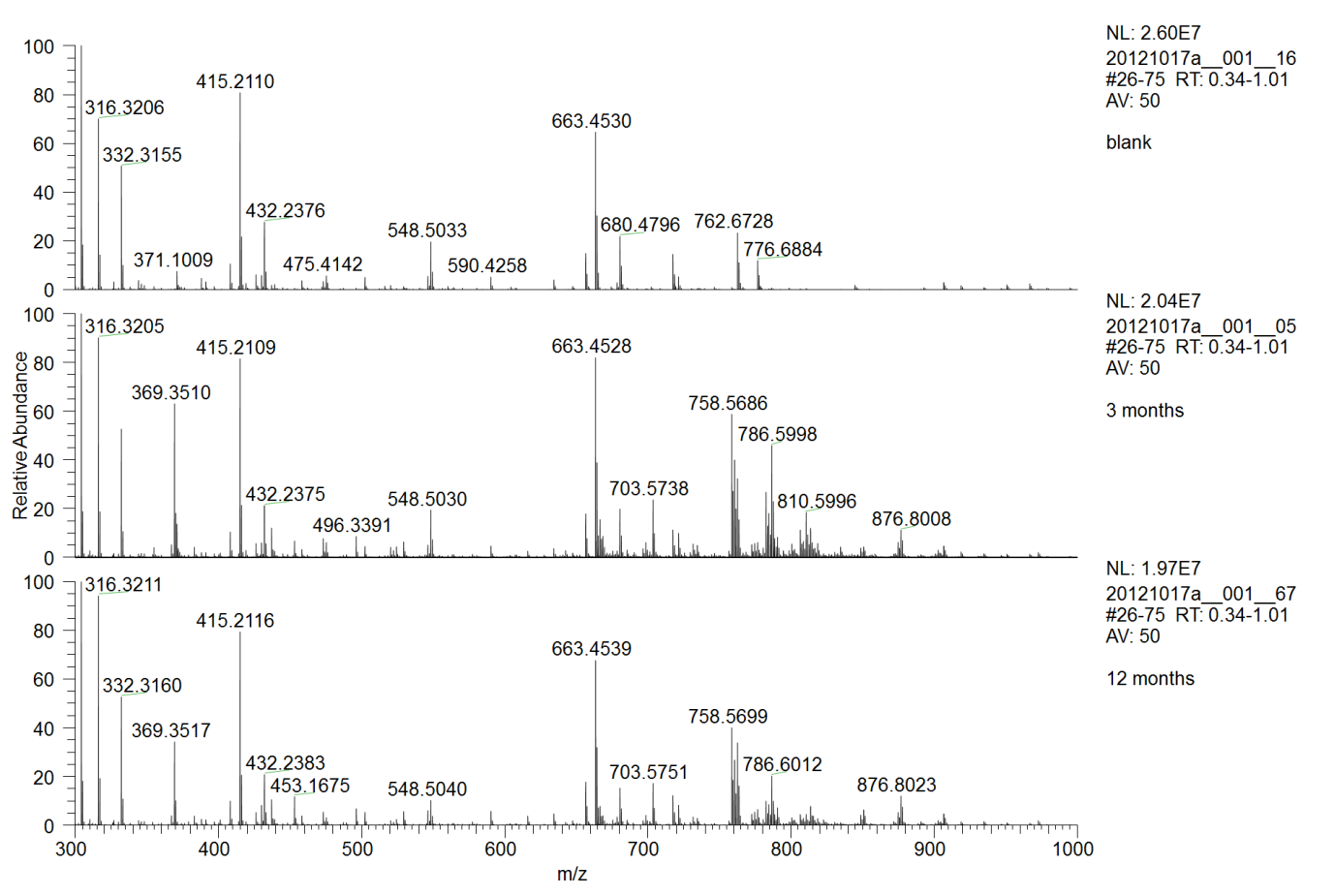


Figure S-1 Average mass spectra obtained by DIHRMS of three different samples type: blank paper (top); dried blood spot of 3 months old infant (middle); and dried blood spot of 12 month old infant (bottom). Each of the depicted spectra is an average of 50 spectra obtained at 1 Hrz by a benchtop Orbitrap MS.


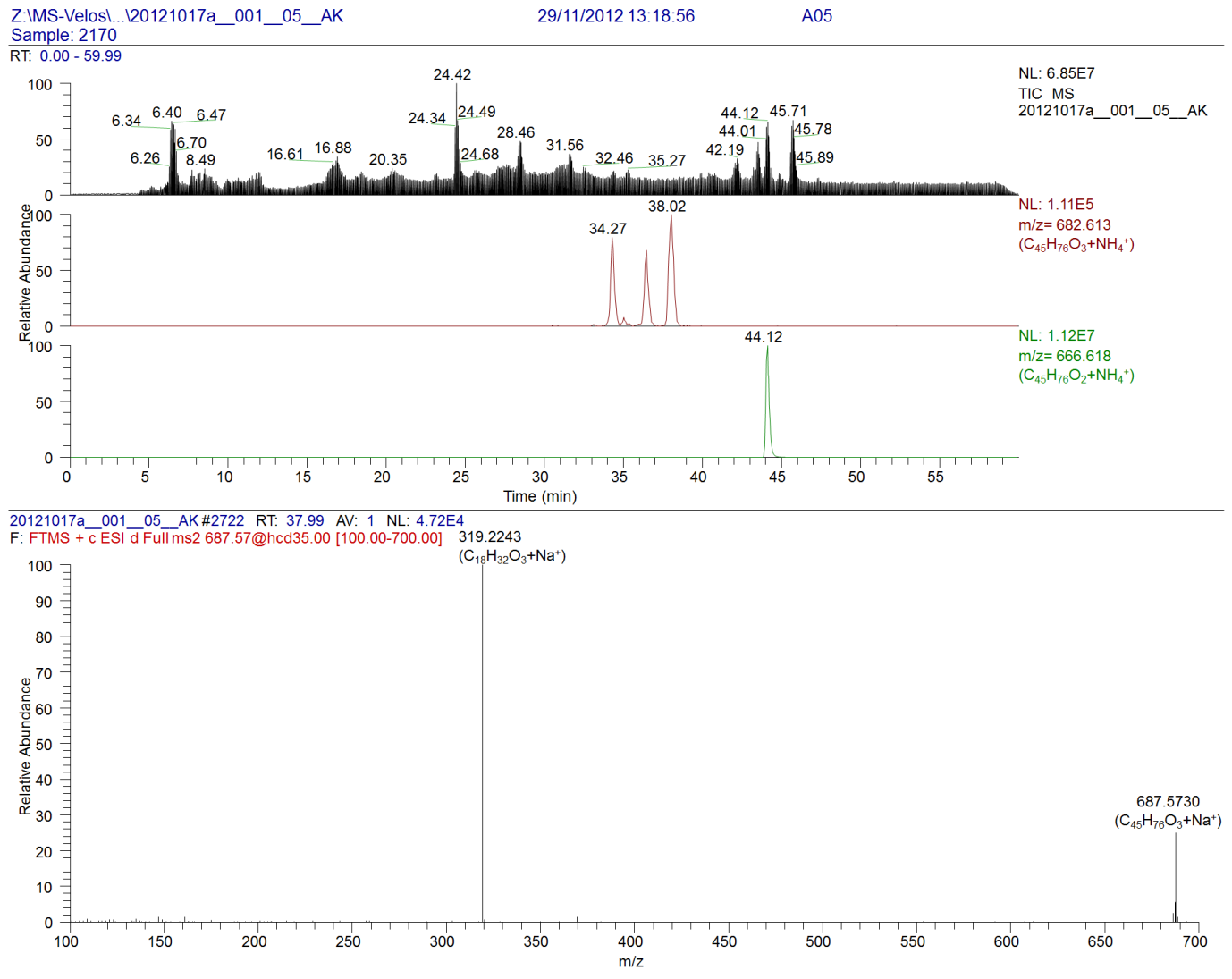


Figure S-2 Example LC-MS chromatogram of a lipid extract from a dried blood spot of 3 months old infant. Top pane: in black: TIC; in red: the selected ion chromatogram for ammoniated oxidized cholesteryl linoleate (m/z 682.613 m/z ± 5 ppm); in green: the selected ion chromatogram for ammoniated cholesteryl linoleate (m/z 668.618 m/z ± 5 ppm). Bottom pane: the MS^2^ spectrum of the sodiated oxidized cholesteryl linoleate showing the sodiated oxidized linoleate as main fragment (see Hutchins et al., 2011).

Figure S-3 OPLS-DA analysis of the lipid profiles obtained from plasma, whole blood or dried blood spots from the same four different healthy adults. This shows that the inter-personal differences in the lipid profile are consistent across the three sample types.

Figure S-4 OPLS-DA analysis of the lipid profiles obtained from dried blood spots from Arterial blood and venous blood of the same five different healthy adults. This shows that the relative differences in the lipid profile are consistent across the two types of blood.
